# Supplementary figures and images for: Genomic evidence of contemporary hybridization between Schistosoma species
Source: PLoS Pathog. 2022 Aug 8;18(8):e1010706. doi: 10.1371/journal.ppat.1010706 (PMC9387932; doi:10.1371/journal.ppat.1010706)

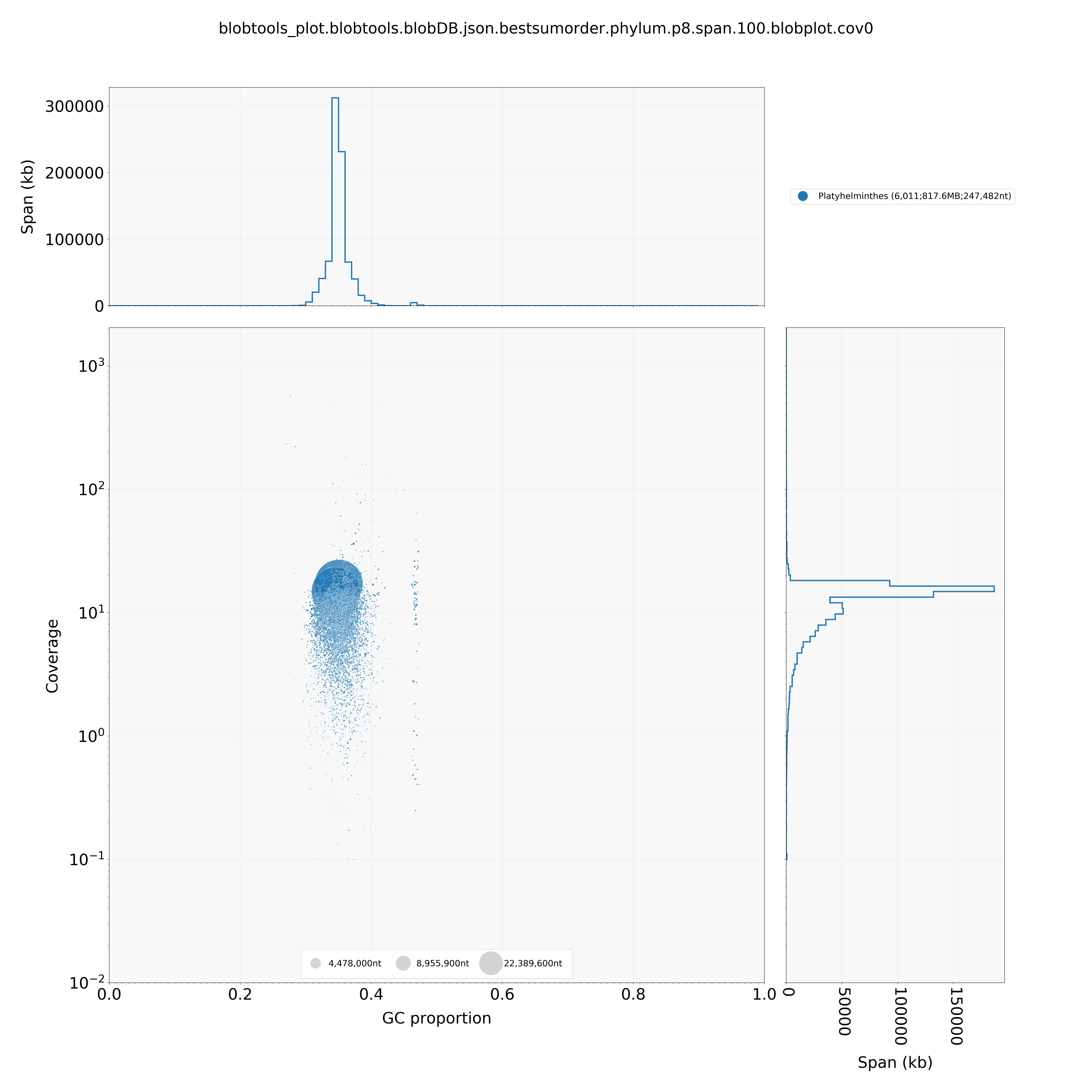

Supplement: S16 Fig — The x-axis values show the GC-proportion for each chromosome and unplaced contig, y-axis values show the coverage of the PacBio subreads mapped to the assembly. Chromosome and unplaced contig sizes are shown by the size of each circle and circles are coloured by taxonomic annotation. (PNG) [file ppat.1010706.s030.png]

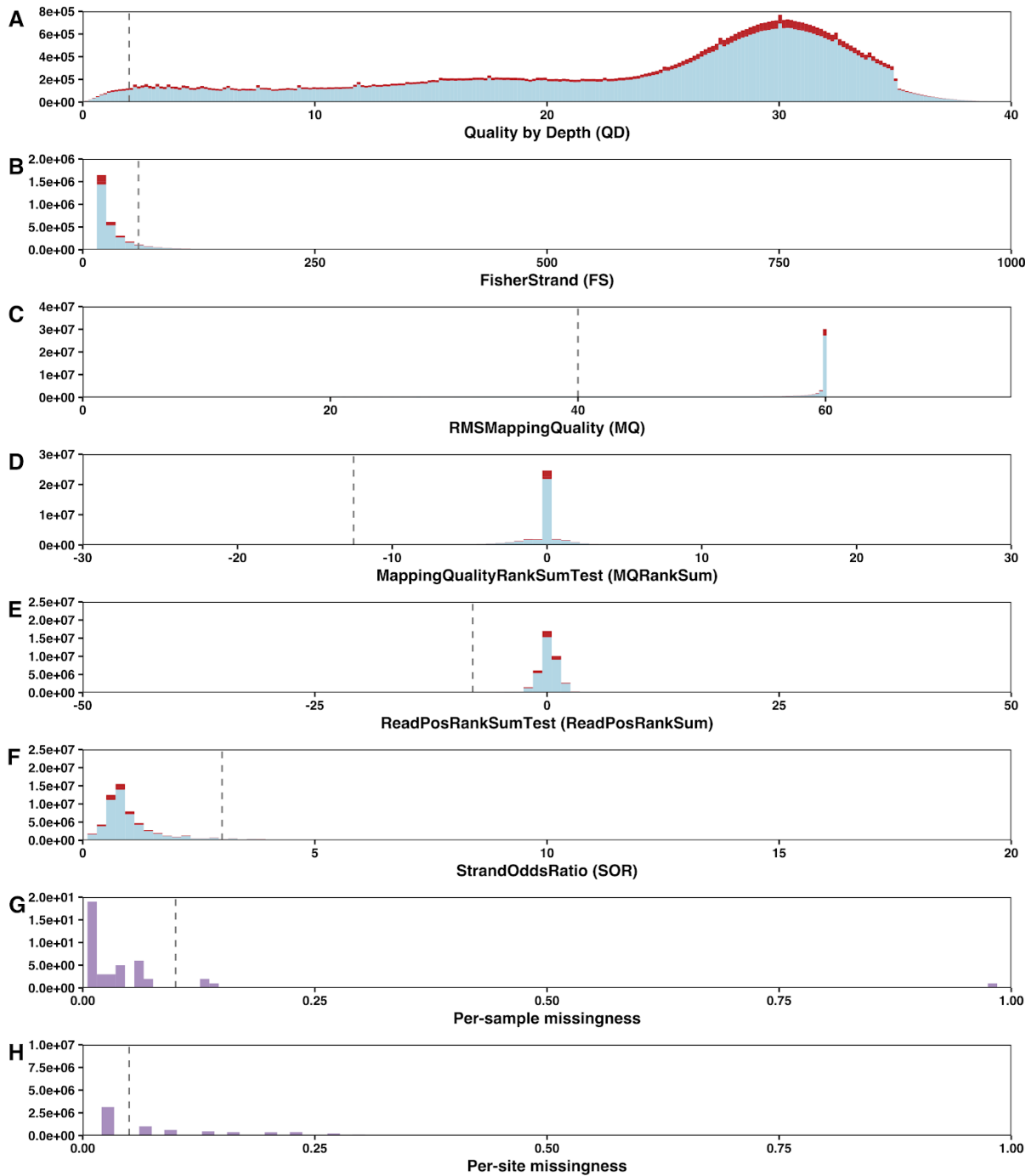

Supplement: S17 Fig — Plots A-F show the frequency distribution of annotation values for 17,395,313 single nucleotide polymorphisms (SNPs) (light blue), indels and mixed sites (SNPs and indels called at the same position for different samples) (red). Plots G-H show the frequency distribution of per-sample missingness (samples with a high rate of per-site variant missingness) and per-site (sites with a high proportion of variant missingness) missingness after filtering using the thresholds in A-F for all remaining variants (purple). Vertical dashed lines show the thresholds applied in the study for removing sites. Samples or sites were removed if they exceeded the thresholds. (PDF) [file ppat.1010706.s031.pdf]
