# Supplementary material for: Genomic evidence of contemporary hybridization between Schistosoma species
Source: PLoS Pathog. 2022 Aug 8;18(8):e1010706. doi: 10.1371/journal.ppat.1010706 (PMC9387932; doi:10.1371/journal.ppat.1010706)
Supplement: Attachment — Submitted filename: Responses to reviewers.pdf [file ppat.1010706.s032.pdf]

Responses to reviewer comments

Reviewer 1

The f3 statistic is intended for populations, not individuals, so its use here is somewhat unconventional. It seems to me that it can indeed function as a test for hybridization when used in this way, but the authors should cite other examples of f3 being applied to individuals, or else explain that this is a novel use of the statistic and state any caveats. Allele frequency in a diploid individual can only be 0, 50%, or 100%, so essentially this is a test for an excess of heterozygous (50% frequency) SNPs among sites known to be fixed between S. curassoni and S. bovis.

Agreed and amended - the manuscript has now been updated with examples of per-individual sample analyses using f_3 and Patterson's D statistics.

Likewise, Patterson's D is being used in a slightly unconventional way here. Patterson's D is much better known as the ABBA-BABA test and it would aid reader comprehension to describe it as such.

Agreed and amended - this test is now also referred to as a ABBA-BABA statistic in the text.

It is not a conventional test for hybridization. It is typically used to distinguish between incomplete lineage sorting and introgression, but that's not what it's doing here, so the authors should remove statements to that effect (e.g. Table 3, Figure 3, and "Genomic signatures of admixture" section). Instead, the authors are (correctly, in my view) rejecting the hypothesis of incomplete lineage sorting AND the hypothesis of introgression in favor if the hypothesis of hybridization. For the hybrids, D is near 0, which (absent any other information) would be consistent with incomplete lineage sorting, and certainly doesn't rule out incomplete lineage sorting, though of course the ABBA BABA results are also consistent with the hybrid ancestries inferred from ADMIXTURE and f3. I can't see how Patterson's D is formally testing any hypothesis here, but if it is the authors should explain the alternative hypothesis that is being ruled out.

We have adjusted our analyses using this metric and have now assessed gene flow from *S. bovis* and *S. curassoni* independently, rather than combined as for our original analyses. The test is now performed using four populations P1, P2, P3, and O, described by the rooted tree (((P1,P2),P3),O). *S. haematobium* was designated as the outgroup population (O), samples to be analysed (newly sequenced non-reference samples) were designated P2, reference populations for *S. curassoni* and *S. bovis* were designated as either P1 or P3 (analysis was performed with populations in each position).

So, now our revised analyses provides values significantly different from 0 in cases of gene flow from either *S. curassoni* or *S. bovis*. In this case it doesn't explicitly function as a test for introgression vs hybridization but rather incomplete lineage sorting vs gene flow. The results of this statistic match the ADMIXTURE/f3/heterozygosity metrics with elevated Patterson's D values when gene flow from *S. curassoni* and *S. bovis* was assessed. Whereas Patterson's D values were only elevated in non-hybrid samples when P3 was the same as the assigned species. This has been clarified within our revised text.

Please provide more details about the F2 backcross inference and definition. Does “F2 backcross” mean a worm that has one purebred parent and one parent that is an F2, or a worm where both parents were derived from an F1 backcross? Specimen BK16_B8_11 is listed as an “F2 backcross” in Table 3, but there is no such designation in the NEWHYBRIDS results presented in Supp Data 8. What is the basis for inferring that it’s an F2 backcross and not an F2? In the text there are repeated mentions of “F2 backcrosses” (plural) but this seems to be the only example. Please clarify.

Apologies, our description in the manuscript was unclear. The F1 backcrosses are now referred to as BC1 (backcrosses of F_1 hybrids to *S. curassoni*). BK16_B8_11 is discussed in more detail in the main text. It’s assignment is ambiguous:

- It contained 18 autosomal ancestry junctions suggesting between three and four generations since admixture.
- NEWHYBRIDS failed to categorise it - describing it as either ‘F2’ or ‘F1 backcross to *S. bovis*’.
- It also had a higher proportion of *S. bovis* ancestry than the F1 hybrids.

From this, we suspect it is a later generation backcross (BC2-BC4) to *S. bovis* but cannot rule out more complex scenarios (mating with other hybrid generations etc). This ambiguity is now clearly stated in the text, and we are grateful to this referee for highlighting this initial lack of clarity.

What does these results mean for the status of these taxa as species? How divergent are they? 309,040 fixed SNPs across a 397 Mb genome is fewer than would be expected between “good” species, but presumably many real SNPs were filtered out. Can the authors estimate the % nucleotide divergence between species based on the sites where reads did reliably align? On the other hand, it seems unlikely that the authors happened to stumble upon one of the first instances of gene flow between these taxa, so perhaps hybridization occurs often but there is selection against hybrids even if they are fertile.

S. bovis and *S. curassoni* are generally considered to be the two most recently diverged species within the Haematobium group (1,2), so a low level of sequence divergence is not surprising. However, 309,040 is much fewer variants than expected, and likely explicable by a number of factors: First is the bias introduced by using a single-species reference assembly, so *S. bovis* specific sequences will not map or map poorly to this assembly and variants in regions of interspecific structural variation would most likely be harder to identify; Second, the identification of fixed variants used a small number of samples and excluded all sites with intraspecies variation or missing variants, further reducing the pool of variants. The goal of these analyses was to identify high-confidence fixed variants not quantify the rate of interspecies variation so this estimate is not a clear reflection of this.

We suspect that due to the above reference bias and limited sample size a quantification of sequence divergence between species would not be accurate and investigations into interspecies variation/genomic definition of taxa. A comparative genomic (multiple reference genomes from multiple species) or pangenomic approach might be more suitable than a single reference approach.

As regards the frequency of hybridization within this system and the selective pressures imposed on them, as we reviewed elsewhere (3), gathering molecular evidence indicates that viable hybridization between species across a broad range of taxa has been, and is ongoing, a potentially common strategy, with both positive and negative selective pressures acting upon the subsequent progeny.

Again we have clarified all these points within our revised text.

Supp Figs 1 and 2 are essentially a single figure split in two, and likewise Supp Figs 3, 4, and 5 are essentially a single figure split into three. The captions are even repeated identically. I understand the need to spread the plots across multiple pages, but it's confusing to have several figures that are described identically. If it has to be several figures, could they be organized by geography or host (e.g. all BK16_B8 samples in the same figure), so there is some justification for the split?

Supplementary figures 1-5 have now been reorganised and split into four groups: 'reference samples', 'adult stage parasites from host BK16_B8', miracidia stage parasites from host BK16_B8' and 'all remaining samples.' Figure legends have been updated accordingly.

Supp Fig 7: MtDNA is inherited as a nonrecombining unit and thus its evolutionary history is fully represented by the phylogeny (part C). Since the variants are linked and not independent, it violates the assumptions of PCA and ADMIXTURE, so parts A, B, and D add nothing to the story and are arguably statistically invalid. A more robust analysis with be to add bootstraps to the phylogeny and/or to use a more powerful phylogeny inference method like maximum likelihood.

A new MtDNA phylogeny has been produced using a maximum likelihood approach. The MtDNA PCA and MtDNA ADMIXTURE analyses have been removed.

Reviewer 2

Please, include page and line numbers in any future submissions. It is highly annoying for a reviewer to comment on a paper without these numbers!

We sincerely apologise for this important typographical oversight here.

Figure 2C. Include bootstrap values

Figure 2C has now been replaced with an unrooted maximum likelihood phylogeny. As bootstrap values would not be clearly visible given the size and branch structure of the tree a larger, bootstrapped version is included as Supplementary Figure 10.

In Introduction: "Recently a child in Niger was found to be infected with an hybrid schistosome derived from two livestock-infective schistosome species.. " --> 'an hybrid' should probably be 'a hybrid'
In Introduction: there is something not quite right about the grammar in this sentence: "whereas more recent hybrid lineages could indicate demographic shifts or anthropogenic environmental changes influence parasite population structures."
In Discussion: 'In the same study, the authors did not find snails shedding S. bovis in Barkedji concluding that S. bovis was mostly likely imported from neighbouring regions.' --> 'mostly likely' should most likely be 'most likely'
In Discussion, conclusion: '..., supporting that indicated by recent population genetic approaches.' Check grammar.

Agreed - all typographical/grammatical issues highlighted have been amended accordingly.

Reviewer 3

My main concerns are below:
*** In the introduction, the authors should probably be slightly more cautious when qualifying schistosomes of hybrids. Most "hybrids" have been identified with only single/few nuclear/mitochondrial markers. Recent studies showed that this can be misleading regarding when the hybridization took place and therefore the true status of hybrid. The authors discussed this point in their introduction and discussion. There are more specific comments below regarding some passages.**

Whist we agree that, particularly for the earliest of published studies using simple morphological and/or molecular techniques, that one could not be entirely sure that those parasites deemed hybrids were in fact hybrids - our very findings here provide further confirmation of the more common ITS/COX-1 typing methods that this referee appears to be referring to. As we discuss this in detail in both our introduction and discussion, we feel it is unnecessary to add additional terminology here. However, if at the editor request, we can refer to early studies indicating 'putative' hybrids if necessary.

*** While I recognize the effort of the authors to carefully assess possible contamination in their samples (p. 13 §2), I still have concerns:**
- The authors focused on the same-species contamination but the inter-species contamination is the most problematic type because leading to identification of hybrids that were actually inter-species mating couples. Nothing is done to address this.

All adult-stage samples were manually inspected prior to DNA extraction to confirm they were individual adult-stage worms and not paired (inter- or intra-species pairings). The text has been updated to make this clearer. Allelic depth screening should be as, if not more effective (as it evaluates heterozygous sites), at detecting inter-species contamination than intra-species contamination

- Looking at the supplementary figure, some samples showed profile that could be problematic (for instance all the SHAE, SINT_01, SMAG_01, SMAT_01) but were not excluded. The criteria/algorithm for exclusion is unclear.

All those samples represent outgroup species (i.e. not *S. curassoni* or *S. bovis*), which functioned as reference samples for each species. The more variable allelic depth profiles illustrated in Supplementary Figure 5 may be a result of inconsistent mapping of individual haplotypes to the *S. curassoni* reference due to the greater phylogenetic distance of these species to *S. curassoni*. Most of these samples were derived from single adult-stage samples that were carefully inspected to make sure they were unpaired/separated worms prior to DNA extraction (4), others were miracidia stage samples (5). Moreover, none of these samples were assessed for evidence of hybridization - they were not the focus of this study, they were purely representative samples for each species (in the case of the non-*S. haematobium* species, they were/are the only available sequence datasets for those species).

The methods section has been updated with clearer exclusion criteria, and now states that the reference samples were excluded from this quality control step.

- There are methods relying on co-segregating SNPs on reads that are robust in estimating number of haplotypes in a sample. For instance [estMOI](https://academic.oup.com/bioinformatics/article/30/9/1292/235531), designed for malaria, could be applied on diploid organism, for which a number of 2 haplotypes would be expected. As this is reference agnostic, analyzing hybrids should not be a problem. Coupled with the current method, this should reveal intra and inter-species contamination. I would suggest to create some test cases with the current data and known pure samples for validating the approach.

We have now included estMOI results in the manuscript, along with the results of the GATK CalculateContamination tool. estMOI did not identify any additional instances of contamination - MOI estimate = 1 in all post-QC samples.

*** **p. 9 l. 13-14**: "child in Niger was found to be infected with an hybrid schistosome derived from two livestock-infective schistosome species": this is based on single nuclear and mitochondrial markers. The authors should be more cautious and probably describe the results only based on the observation, especially knowing that no active hybridization has been identified in Niger at the genome level ([Platt et al. 2019](https://pubmed.ncbi.nlm.nih.gov/31251352/)). Here is a suggested rephrasing of the sentence: "child in Niger was found to be infected with a schistosome displaying markers derived from two livestock-infective schistosome species (...) futher suggesting ...".**

The above correction has been made.

*** **p. 9 l. 22-23**: "hybridization between *S. bovis* and *S. haematobium* appears to have facilitated a 2013 outbreak of schistosomiasis in Corsica": a recent paper ([Rey et al.](https://journals.plos.org/plospathogens/article/file?id=10.1371/journal.ppat.1009313&type=printable)) reclassified the "hybrid" Corsican sample as a *S. haematobium*. The authors should therefore be cautious about considering this sample as hybrid. This also applies to **p. 10 l. 20-23**.**

We have updated the text to reflect the controversy surrounding these samples.

*** **p. 10 l. 4**: "Hybrid schistosomes" should be "Schistosomes from possible hybridization event".**
*** **p. 10 l. 9**: "is likely" should be "could".**
*** **p. 10 l. 20**: please see previous comment. Again, caution is needed.**
*** **p. 11 l. 3-4**: "hybrid lineages are present" should be "hybrid lineages could be present".**
*** **p. 19 l. 9**: "readily": the authors should remove the adverb because the constraints leading to hybridization are unknown. While time since divergence can indeed explain how easy it is for these two species to hybridize, scarcity of partners within the host may in fact be the reason of the hybridization that may not occur otherwise.**
*** **p. 20 l. 5**: "hybrids combinations have been identified to infect humans": same remark as for p. 9 l. 13-14.**

The above text, where appropriate, corrections have been made.

* **p. 6**: "spp" should be "spp." (period missing).
* **p. 10 l. 14**: "from infected children with Niger": this formulation sounds strange. Should this be "from infected children in Niger" ("with" replaced with "in")?

The above typographical/grammatical corrections have been made.

* **p. 12 l. 8**: "most recent": the version v7 is no longer the most recent as v9 is now available. The authors should replace this with "recent" or "version 7" (removing the "(v7)" in this case).

The genome assembly section results have been updated to use version 9 of the *S. mansoni* assembly. Additionally, Table 1 has been updated with the most recent versions of the *S. haematobium* and *S. japonicum* assemblies. This has again been clarified within our revised text.

* **p. 25 and 26**: Genotyping and sequencing sections: the authors should provide the quantity of DNA used for genotyping and preparing the sequencing libraries. What was the kit used for library preparation?

More detailed protocols for library preparation have now been included in the methods section. Input DNA was not quantified prior to genotyping and information on the quantity of input DNA was not available from Wellcome Sanger Institute DNA pipelines.

* **p. 27 l. 20**: "S. curassoni" should be "*S. curassoni*" (italic missing).
* **p. 27 last l.**: "S. bovis" should be "*S. bovis*" (italic missing).
* **p. 43**: "published datasets." should be "published datasets)." (closing parenthesis missing).
* **Fig. 1 & 2**: letters associated to each panel should be either lower case or upper case for consistency.

Again these typographical errors have been amended and we thank this reviewer for spotting them.

* **Supp. Fig. 3 to 5**: only 3 out of 4 samples are tagged as excluded. I suspect that this is due to the removed sample showing low genotyping rate. This should be clarified in the text.

Main text has been clarified to differentiate between the three samples excluded due to contamination and the one sample removed due to low genotyping rates.

References

1. Jones BP, Norman BF, Borrett HE, Attwood SW, Mondal MMH, Walker AJ, et al. Divergence across mitochondrial genomes of sympatric members of the Schistosoma indicum group and clues into the evolution of Schistosoma spindale. Sci Rep. 2020 Feb 12;10(1):2480.

2. Zajac N, Zoller S, Seppälä K, Moi D, Dessimoz C, Jokela J, et al. Gene Duplication and Gain in the Trematode Atriophallophorus winterbourni Contributes to Adaptation to

Parasitism. Genome Biol Evol [Internet]. 2021 Mar 1;13(3). Available from: http://dx.doi.org/10.1093/gbe/evab010

3. King KC, Stelkens RB, Webster JP, Smith DF, Brockhurst MA. Hybridization in Parasites: Consequences for Adaptive Evolution, Pathogenesis, and Public Health in a Changing World. PLoS Pathog. 2015 Sep;11(9):e1005098.

4. International Helminth Genomes Consortium. Comparative genomics of the major parasitic worms. Nat Genet. 2019 Jan;51(1):163–74.

5. Platt RN, McDew-White M, Le Clec'h W, Chevalier FD, Allan F, Emery AM, et al. Ancient Hybridization and Adaptive Introgression of an Invadolysin Gene in Schistosome Parasites. Mol Biol Evol. 2019 Oct 1;36(10):2127–42.
